# Lead poisoning due to bullets lodged in the human body

**Published:** 2012-09-30

**Authors:** Juan Bernardo Gerstner Garcés, Rafael Ignacio Manotas Artuz

**Affiliations:** 1 Instituto de Enfermedades Osteoarticulares, Centro Medico Imbanaco de Cali (Colombia ); 2 Department of Orthopaedics and Traumatology. Universidad del Valle. Cali, Valle, Colombia.

**Keywords:** Lead poisoning, wounds, gunshot, feet

## Abstract

With the increased violence and use of firearms in Colombia, we may see more cases of lead poisoning in our environment, and must be prepared to diagnose and treat them. Subtle signs and symptoms as unexplained anemia, gastro-intestinal discomfort and abdominal cramps, as well as severe signs such as changes in behavior and neurological status, nephropathy, and unexplained death, may be associated with a history of gunshot wounds and bullets in the human body. We must offer the patient knowledge and management strategies of pathology.

## Abstract (es)

Con el aumento de la violencia y el uso de armas de fuego en Colombia, es posible que veamos más casos de intoxicación por plomo en nuestro medio, y debemos estar preparados para diagnosticarlo y tratarlo. Síntomas y signos sutiles como anemias no explicadas, malestar gastro-intestinal y calambres abdominales, y severos, como cambios en la conducta y el estado neurológico, nefropatía y muerte inexplicable, pueden estar asociados con un antecedente de heridas por arma de fuego y proyectiles en el cuerpo humano, y debemos ofrecer al paciente conocimiento y estrategias para el manejo de la patología.

## Introduction

Lead intoxication (saturnism) caused by bullets or projectiles lodged in the human body is an under-diagnosed condition, that can be fatal if not recognized^1^ ^-^ ^3^. Projectiles lodged in the joints or pseudo-cysts are more likely to develop this complication due to contact with synovial fluid. However, patients with projectiles elsewhere may also be at risk of developing intoxication^3^ ^-^ ^5^.

Few lead poisoning cases secondary to firearm wounds have been reported in the literature. The more frequent cases have been those of patients with bullets lodged in the spine, hands and knees. Subtle findings, such as the appearance of an unexplained anemia, abdominal cramps, kidney, or neurological impairment in patients with a history of projectile wounds may suggest the diagnosis of lead poisoning^1^ ^,^ ^6^. A hyper-metabolic stress factor, such as an infection, endocrinopathy or alcoholism could be a trigger.

Among the various diagnostic studies available, the analysis of stable-isotope dilution (SID) spectrometry and lead serum levels may be the most reliable. It is important to use chelation therapy before any surgical intervention for reducing the mobilization of lead in the bones during or after the surgical procedure^7^ ^-^ ^10^.

The management of projectile fragments or shrapnel in the human body is chronologically divided into 3 phases: acute, in which the management is based on the application of the ATLS protocol and, depending on the severity of soft tissue injury, washing and wound debridement. The sub-acute phase in which one proceeds to the extraction of the foreign body in cases of local or systemic symptoms. Finally, the late phase is usually of asymptomatic fibrotic changes in which the surgeon usually does not intervene.

The purpose of this article is to review the literature published to date about the physiopathology, diagnosis and treatment of secondary lead poisoning to firearm projectiles in an intra-articular location. We also want to illustrate our recent experience with the management of two cases that were presented at our institution for patients with lead poisoning.

### Physiopathology

Lead fragments lodged in joints undergo a process of disintegration; however, the majority of patients with projectiles lodged in the body are not treated for systemic absorption. The sequence of events by which lead is systematically mobilized is unknown, the presence of radiopaque material in the walls of the pseudo-cyst are presumably some form of lead has been identified by means of electron microscopy and centrifugation of samples of synovial fluid.

The mechanism is not entirely clear, but apparently the lead in the walls of the projectile is initially released in a soluble form which is directly absorbed into the bloodstream, later it precipitates to another insoluble form which is trapped and phagocytized from the pseudocyst by macrophage. This process is similar to that occurring with lead dioxide (PbO_2_) which is inhaled in the form of micro particles by workers handling lead and is later withdrawn from the respiratory tract by macrophages with subsequent cell death and its extracellular deposition^10^.

The intra-articular presence of lead particles could trigger an inflammatory arthropathic process concomitant with increased vascularity and inflammatory cells which would explain systemic absorption from the joint.

After absorption, lead is distributed mainly in three compartments: in the first place it circulates in the blood bound to red blood cells, 95% of the lead is attached to the erythrocyte; later it is distributed to soft tissues, such as the liver, bone marrow, kidneys and central nervous system. Finally, after 1-2 months the lead spreads to the bones where it can stay for up to 30 years. Interfering with calcium metabolism by binding to calmodulin, inhibits the sodium-potassium pump (Na-K-ATPase) and activates the protein kinase which ultimately triggers neurotransmission disorders and vascular tone. In the kidney it interferes with the conversion to the active form of Vitamin D and glomerulopathy which eventually leads to a selective proteinuria.

### Diagnosis

Clinical symptoms are the mainstay for the diagnosis of lead poisoning. Unexplained abdominal pain, motor neuropathy in the extremities, nephropathy, poly-arthralgia and neurological disorders are among the most common symptoms. Burton edging, a dark line between the base of the teeth and gums, is one of the clinical signs of lead poisoning.

Laboratory tests are important, such as the CBC with findings of anemia that may be normochromic or hypochromic, normocytic or microcytic, basophilic stippling that although it is not pathognomonic is very characteristic of lead poisoning.

The presence of β2 microgobulin in the urine serves as an early marker of kidney damage and semen analysis can be altered both by the number and the shape of the sperm.

Sampling levels of lead in the serum and urine is relevant since concentrations around 30 µg/dL have shown deleterious effects on the central nervous system and kidneys^7^ ^-^ ^10^. Urinalysis for determining delta aminolevulinic acid (ALA-U) (normal value: 6 mg/dL) and zinc protoporphyrin levels (normal value: 75 µg /dL) contribute to the diagnostic process^2^ ^,^ ^6^ ^,^ ^9^.

Clinical manifestations directly correlate with the levels of lead in the blood. Lead encephalopathy occurs with levels above 80 ug/dL, cognitive impairment with 50 µg/dL, nephropathy with 40 µg/dL and peripheral neuropathy with 20 µg/dL. Some conditions that cause metabolic stress such as infection, endocrinopathy and alcoholism may be precipitating factors of the condition^8^ ^-^ ^10^.

For the initial evaluation of the patient, it is usually sufficient to perform basic radiographs of the compromised extremities as evidence of the presence of projectiles or fragments from firearms with multiple loads. Similarly, Computerized Axial Tomography (CAT) contributes to finding the exact location of the projectile especially in cases of injury to the spinal cord, thorax and abdomen. Ultrasound has proven to be a useful tool for a dynamic evaluation and for establishing the relationship of the shrapnel or projectiles with the joints, tendons, and vascular structures.

Techniques using fluoroscopy, computerized navigation and Iso-C 3D (an instrument that provides for three-dimensional intra-operative reconstruction) have provided for the performance of surgical procedures with greater ease and accuracy in recent years. 

### Treatment of secondary lead poisoning from projectiles lodged in the human body

The patient's treatment regimen consists of multidisciplinary medical and surgical management. Multiple factors such as the manipulation of bone and soft tissue cause a redistribution of the lead and an increase in blood levels, for that reason in patients about to undergo surgery chelation therapy prior to the procedure is recommended. Similarly, if the serum lead level is greater than 60 µg/dL or if there are clinical manifestations as previously described in Table 1, chelation therapy should be initiated^7^ ^,^ ^10^.

**Table 1:**
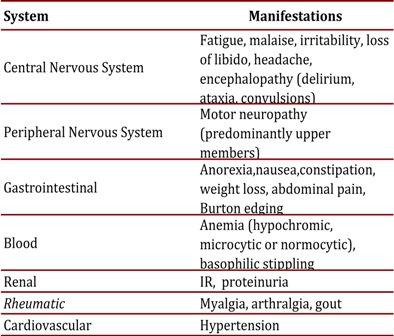
Clinical manifestations of lead poisoning systems

A principle in toxicology is to remove the source of toxicity from the patient; however, sometimes the extraction of projectiles or fragments may be technically difficult and cause damage to vital structures during the procedure. It is therefore important to assess the risk-benefit at the time of making the decision to extract the firearm projectiles in the body and reserve surgical management for those cases of patients with symptoms of lead toxicity, especially in those cases with projectiles lodged in joints or in the spinal cord in contact with spinal fluid.

### Therapy and chelating agents used are as follows:

a. Edetate Disodium-Calcium-(Ca-EDTA): 30-50 mg/kg/day administered intravenously during 8 hours for 6 days. In cases of lead encephalopathy and patients with blood lead levels greater than 100 µg/dL Dimercaprol should be added. 

b. Dimercaprol (BAL): 3-5 mg/Kg/dose. Start 1 hour before the EDTA. Administer IM every 4-6 hrs for 5 days.

c. Dimercaptosuccinic acid (DMSA): 10 mg/kg/dose orally administered every 8 hours for 6 days. Continue every 12 hrs for 2 weeks.

Therapy should be monitored with lead levels in the urine to evaluate the total excretion of lead per day of treatment.

### Case 1

The patient was a forty-three year old male, with evolving malaise and progressive weakness over a four year period in all 4 limbs with episodes of partial improvement and frequent relapse. He was evaluated at our institution for exacerbation of symptoms. History of Guillain-Barre dating to 16 years prior, controlled hypertension, chronic renal insufficiency, stage IV, and chronic anemia syndrome. There was a history of a firearm wound to a foot 24 years earlier with orthopedic management. When examined he presented with muscular weakness predominantly in the upper limbs and hyporeflexia without involvement of the cranial nerves. Blood profile: Hemoglobin: 8.3 g/L, Hematocrit: 25. Microcytosis. Na: 135 K: 5.1. C3-C4: normal, VDRL: No reagent.

**Figure 1:**
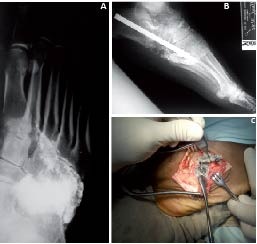
1.caso A. Oblique Projection Radiography: Fragments of lead level Chopart and Lisfranc joints. B. X-ray of the foot. Oblique. Autografts column and osteosynthesis fixing the sidebar. C. Lateral approach to the midfoot. Extraction of lead fragments

Blood smear (-). HIV (-). Rated by Internal Medicine and Neurology with a diagnostic impression of: Viral myelopathy, Guillain-Barré sequelae. Radiographs show presence of multiple fragments of lead in the sidebar of the foot with partial destruction of the Cuboids (Fig. 1A).

We considered the possibility of lead poisoning. Blood lead levels were taken and reported as 62 ug / dL (Normal: 0-9.9). Starting chelation therapy with EDTA: administered 50 mg/kg/day.

After chelation therapy, the patient was taken to surgery for removal of the bullet material and midfoot reconstruction (Figs. 1B-C).

### Case 2

**Figure 2:**
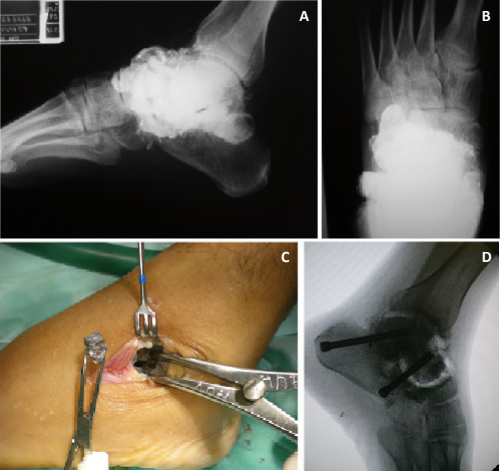
Case 2: A. AP radiograph. Commitment severe subtalar and Chopart joint by gun fire. B. AP radiograph. Commitment severe subtalar and Chopart joint by gun fire. C. subtalar approach. Removing Projectiles and intraarticular fragments lead. D. Lateral Ankle radiograph. Subtalar arthrodesis with compression screws. Liberation Talo-Navicular joint and autografts

The patient was a 38 year old male with a 3-year history of evolving paroxysmal abdominal pain, postprandial emesis and weight loss. Medical management with partial improvement reported. Progress accompanied by exacerbation of symptoms, bizarre behavior, aggression and seizures. The patient was initially wounded 15 years ago with a shot to the foot. Smoking and drinking to intoxication was reported. Physical examination showed decreased muscle strength with predominantly at C5 -T1 levels. Achilles hyporeflexia. The blood count and peripheral blood smear showed hypochromic microcytic anemia. Lead Levels were: 90.3 µg/dL. Radiograph of foot evidences multiple metal fragments at the midfoot joints. Osteoarthritis of the calcaneocuboid joint, Talo-navicular and subtalar (Fig. 2A). Diagnosis of blood poisoning, starting chelation with EDTA (50 mg/kg/day) for 7 days ago. It is taken to surgery for removal of projectiles lodged and hindfoot arthrodesis (Figs. 2B-D).

## Discussion

Lead poisoning in continues to be a rare phenomenon, but given the significant number of patients treated for firearm wounds it should be suspected. If not ruled out through diagnostic procedures, initiation of appropriate treatment should be commenced.

Manifestations in the musculoskeletal, renal, central nervous system, gastrointestinal and hematopoietic systems have been shown to be the most frequently compromising to the patient´s health to differing degrees of severity.

Young patients suffering diffuse symptoms from unexplained causes, such as unexplained anemia, recurrent and diffuse gastrointestinal pain, polyarthralgias and neurological involvement must be analyzed carefully for a history of traumatic injuries that may explain the presence of lead in the body. It is important to take lead levels in the blood and urine as a diagnostic test and consider that some conditions that cause metabolic stress, such as infection, endocrinopathy and alcoholism may be precipitating factors. 

Medical management prior to surgery is mandatory as particles can be removed from the bloodstream and/or synovial fluid during the procedure that could lead to increased plasma levels and produce a lead encephalopathy.

Multi-disciplinary management should be initiated in all cases, along with securing a proper history, and recognizing the signs and symptoms of lead poisoning are all fundamental elements for the effective treatment of this phenomenon. 
